# Loss of Mitochondrial AAA Proteases AFG3L2 and YME1L Impairs Mitochondrial Structure and Respiratory Chain Biogenesis

**DOI:** 10.3390/ijms19123930

**Published:** 2018-12-07

**Authors:** Jana Cesnekova, Marie Rodinova, Hana Hansikova, Jiri Zeman, Lukas Stiburek

**Affiliations:** Department of Pediatrics and Adolescent Medicine, First Faculty of Medicine, Charles University in Prague and General University Hospital in Prague, 12808 Prague, Czech Republic; tesarovajaniky@gmail.com (J.C.); marie.rodinova@lf1.cuni.cz (M.R.); hhansikova@seznam.cz (H.H.); jzem@lf1.cuni.cz (J.Z.)

**Keywords:** mitochondria, protease, AFG3L2, YME1L, AAA complex

## Abstract

Mitochondrial protein quality control is crucial for the maintenance of correct mitochondrial homeostasis. It is ensured by several specific mitochondrial proteases located across the various mitochondrial subcompartments. Here, we focused on characterization of functional overlap and cooperativity of proteolytic subunits AFG3L2 (AFG3 Like Matrix AAA Peptidase Subunit 2) and YME1L (YME1 like ATPase) of mitochondrial inner membrane AAA (ATPases Associated with diverse cellular Activities) complexes in the maintenance of mitochondrial structure and respiratory chain integrity. We demonstrate that loss of AFG3L2 and YME1L, both alone and in combination, results in diminished cell proliferation, fragmentation of mitochondrial reticulum, altered cristae morphogenesis, and defective respiratory chain biogenesis. The double AFG3L2/YME1L knockdown cells showed marked upregulation of OPA1 protein forms, with the most prominent increase in short OPA1 (optic atrophy 1). Loss of either protease led to marked elevation in OMA1 (OMA1 zinc metallopeptidase) (60 kDa) and severe reduction in the SPG7 (paraplegin) subunit of the m-AAA complex. Loss of the YME1L subunit led to an increased Drp1 level in mitochondrial fractions. While loss of YME1L impaired biogenesis and function of complex I, knockdown of AFG3L2 mainly affected the assembly and function of complex IV. Our results suggest cooperative and partly redundant functions of AFG3L2 and YME1L in the maintenance of mitochondrial structure and respiratory chain biogenesis and stress the importance of correct proteostasis for mitochondrial integrity.

## 1. Introduction

Mitochondria are essential cellular organelles, whose function and integrity is ensured by action of quality control mechanisms, including maintenance of mitochondrial proteostasis by proteases and chaperones, ongoing mitochondrial fusion and fission events, and removal of severely damaged mitochondria by mitophagy [1,2,3]. Mitochondria harbor several proteolytic complexes with distinct substrate specificities. These complexes reside within the various mitochondrial subcompartments and carry out essential functions required for maintenance of correct mitochondrial proteostasis. Two ATP (adenosine triphosphate)-dependent proteolytic complexes, the m- and i-AAA (ATPases associated with various cellular activities) proteases, are found within the inner membrane of mitochondria. They extend their proteolytic domains to the opposite sides of the inner membrane. While i-AAA protease carries proteolysis at the intermembrane face, the m-AAA complex is known to function proteolytically at the matrix side of the membrane. The i-AAA protease is known to exist only as a homo-oligomer of YME1L subunits. The human m-AAA protease is found as two isoenzymes in human cells: a homo-oligomer of AFG3L2 subunits and hetero-oligomer of AFG3L2 and SPG7 [4]. Mitochondrial AAA proteases are known to mediate various processes from protein quality control to regulation of mitochondrial dynamics and respiratory chain biogenesis [1,4,5,6]. The i-AAA protease subunit YME1L, along with the inner membrane protease OMA1, is involved in the proteolytic processing of the dynamin-like GTPase (guanosine triphosphatase) OPA1, which is central for mitochondrial fusion. Besides its function in the regulation of mitochondrial dynamics, YME1L is involved in the proteolytic processing of non-assembled Cox4, NDUFB6, and ND1 subunits of the mitochondrial respiratory chain [7,8]. Remaining known mammalian YME1L substrates include Tim17A, a stress-regulated subunit of the TIM23 mitochondrial protein import complex and OMA1 metallopeptidase [9,10]. We have shown the human LACE1 ATPase as a possible YME1L adaptor in the proteolysis of complex IV subunits [11]. Similarly, AFG3L2 was implicated in the processing of Cox1 and MT-ATP6 mitochondrially-encoded respiratory chain subunits [12,13]. Recently, both m-AAA protease subunits AFG3L2 and Spg7 were shown to degrade the unassembled EMRE subunit of the mitochondrial calcium uniporter complex [14]. Very recently, AFG3L2 was shown to mediate proteolytic processing of OMA1 metallopeptidase [15], and Spg7 was identified as a key component of the mitochondrial permeability transition pore (PTP) [16]. Surprisingly, the metalloprotease activity of Spg7 was shown to be dispensable for its function in mitochondrial permeability transition [16]. Mutations in AFG3L2 and SPG7 are associated with autosomal dominant spinocerebellar ataxia (SCA28) and autosomal recessive hereditary spastic paraplegia (SPG7), respectively. Concurrent AFG3L2 and SPG7 mutations were recently shown to be associated with syndromic parkinsonism and optic atrophy [17]. Homozygous YME1L1 mutation was shown to be associated with mitochondriopathy, optic atrophy, and mitochondrial network fragmentation [18]. Recently, the loss of YME1L in a mouse model was shown to lead to ocular dysfunction and spinal axonopathy [19]. In the present study, we focused on the characterization of possible functional overlap and cooperativity of AFG3L2 and YME1L in the regulation of mitochondrial dynamics and respiratory chain biogenesis. We demonstrate that loss of AFG3L2 and YME1L results in reduced cell proliferation, fragmentation of mitochondrial reticulum, altered cristae morphogenesis, and defective respiratory chain biogenesis. Our results reveal cooperative and partly redundant involvement of mitochondrial AAA proteases in the maintenance of mitochondrial structure and respiratory chain biogenesis.

## 2. Results and Discussion

### 2.1. Knockdown of AFG3L2 and YME1L Leads to Increased Accumulation of Complex I, IV and V Subunits

**Figure 1:**
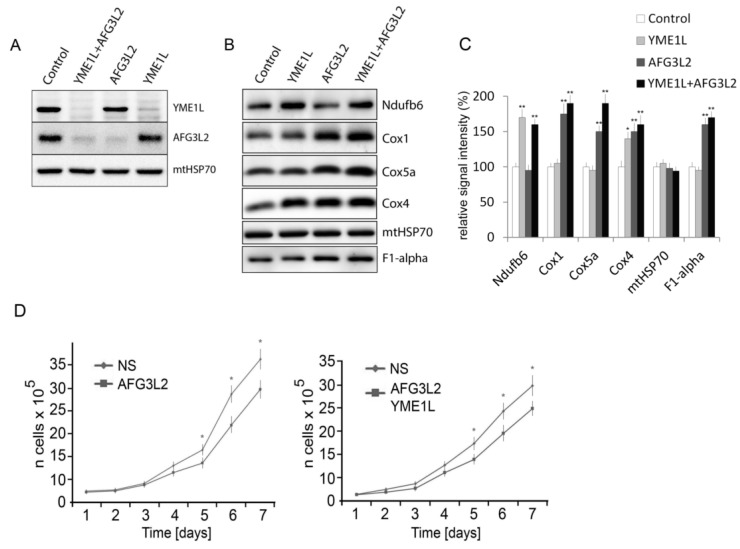
Knockdown of AFG3L2 and YME1L by shRNA leads to increased accumulation of complex I, IV, and V subunits and reduced growth rate of the cells. (**A**) Knockdown (KD) of YME1L, AFG3L2, or their combination by stable shRNA expression leads to marked reduction of corresponding protein levels. Whole-cell lysates from shRNA knockdown cell lines were immunoblotted with antibodies to YME1L, AG3L2, or mtHSP70. The control cell line was prepared by transfecting HEK293 cells with the non-silencing (scrambled) shRNAmir vector. (**B**) Altered accumulation of respiratory chain subunits in the KD cells. Whole cell lysates were immunoblotted with antibodies to Ndufb6, Cox1, Cox5a, Cox4, mtHSP70, and F1-alpha. (**C**) The quantification of western blot signal from B by densitometric analysis. The values are shown as mean ± SD. * *p* < 0.05; ** *p* < 0.01. Western blot images representative of three independent experiments are shown. (**D**) Stable KD cells were seeded in six-well plates at 5 × 10^4^ cells per well and cultured in DMEM (Dulbecco’s Modified Eagle Medium) containing 1 ug/mL puromycin. Viable cells were counted every 24 h for a total of 7 d and the values (mean ± SD) were plotted. * *p* < 0.05.

With the aim of characterizing the functional overlap and cooperativity of AFG3L2 and YME1L in mitochondrial biogenesis and proteostasis, we generated stable HEK293 (Human embryonic kidney) cell lines with shRNA-downregulated expression (Open Biosystems) of AFG3L2 (NM_006796.1) and YME1L (NM_014263), as well as both AFG3L2 and YME1L simultaneously [7]. A control cell line was prepared by transfecting HEK293 cells with commercially available scrambled (non-silencing) shRNAmir-containing expression vector RHS436 (Open Biosystems) [20]. Western blot analysis with antibodies to AFG3L2 and YME1L confirmed that all three produced cell lines contained less than 10% of residual target protein levels (Figure 1A).

We have previously demonstrated that YME1L protease affects the stability of Cox4 and Ndufb6 respiratory chain subunits by mediating their proteolytic degradation [7]. However, not much is known of AFG3L2 protease involvement in oxidative phosphorylation system biogenesis and of the possible substrate overlap or cooperation between m-AAA and i-AAA complexes in this process [21]. We have therefore performed SDS-PAGE (sodium dodecyl sulfate polyacrylamide gel electrophoresis) western blotting screen using mitochondrial fractions isolated from the respective knockdown (KD) cell lines to identify affected respiratory chain and ATP synthase subunits. The screen was limited by the number of available antibodies, but we were able to identify several affected OXPHOS (oxidative phosphorylation system) subunits in the knockdown cells (Figure 1B). In YME1L KD mitochondria, both Ndufb6 and Cox4 subunits were found to be increased, which is consistent with our previous report [7]. On the other hand, western blots of mitochondrial fraction from AFG3L2 KD cells revealed increased levels of Cox1, Cox4, and Cox5a subunits, as well as of the ATP synthase subunit F1-alpha. Finally, mitochondria from the double knockdown YME1L/AFG3L2 cells showed markedly increased levels of Ndufb6, Cox1, Cox4, Cox5a, and F1-alpha subunits (Figure 1B).

We have previously shown that loss of YME1L results in the reduced growth rate of the cells, attributed either to their reduced apoptotic resistance or diminished respiratory capacity [7]. To assess the overall effects of AFG3L2 and YME1L knockdown on cell viability, we examined the growth rates of AFG3L2 and AFG3L2/YME1L knockdown cells over a time course of 7 days. We found significant growth retardation associated with loss of function of either AFG3L2 or AFG3L2 and YME1L (Figure 1C). This could be attributed to hampered mitochondrial bioenergetics or increased sensitivity of the cells to apoptosis [7].

### 2.2. Loss of AFG3L2 and/or YME1L Leads to Mitochondrial Fragmentation and Cristae Depletion and Disorganization

**Figure 2:**
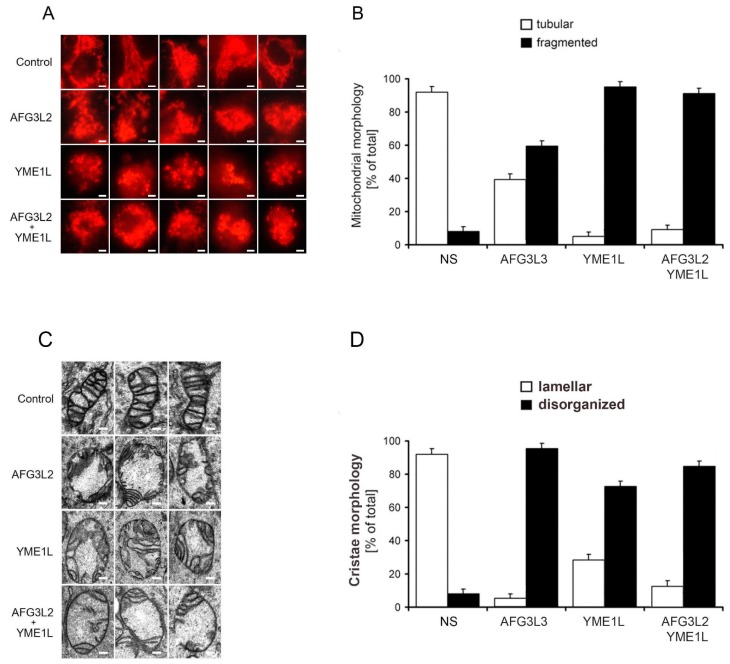
Loss of AFG3L2 and/or YME1L leads to mitochondrial fragmentation and severely disorganized and attenuated cristae architecture. (**A**) AFG3L2, YME1L, and AFG3L2/YME1L knockdown cells exhibit markedly fragmented mitochondrial reticulum. Cells were analyzed using a Nikon Diaphot 200 inverted microscope equipped with an Olympus DP50 camera. Bar, 10 μm. (**B**) The quantification of mitochondrial network morphology in control and KD cells. Cells containing tubular (white bars) or fragmented (black bars) mitochondria were counted in a double-blind manner. More than 100 cells were scored per experiment. (**C**) AFG3L2, YME1L, and AFG3L2/YME1L KD cells exhibit markedly altered and attenuated cristae architecture. The cells were incubated in PBS containing 2% potassium permanganate for 15 min, washed with PBS, and dehydrated with an ethanol series. They were then embedded in Durcupan Epon, sectioned by microtome to thicknesses ranging from 600 to 900 Å, and stained with lead citrate and uranyl acetate. The sections were viewed with a JEOL JEM-1200 EX transmission electron microscope. Bars correspond to 200 nm. (**D**) The quantification of cristae morphology in KD cells. Approximately 50 sections of individual cells were scored in a double-blind manner. Error bars correspond to SD from the mean.

Dynamic mitochondrial network fragments under stress conditions allows the segregation of damaged mitochondria [22]. Regarding proteolytic regulation, OPA1 GTPase can be considered as a central regulator of mitochondrial dynamics and cristae morphogenesis [15,23]. The proteolytic processing of eight human OPA1 splicing isoforms, carried out by YME1L and OMA1 proteases, represents a major checkpoint of mitochondrial fusion/fission events. The i-AAA protease YME1L cleaves OPA1 constitutively, leading to balanced accumulation of long and short OPA1 protein forms, which supports fused network. In contrast, the cleavage of OPA1 by OMA1 metalloprotease is mostly stress-induced, leading to accumulation of soluble short OPA1 forms, inhibited fusion, and mitochondrial fragmentation [24]. We have previously reported that loss of YME1L leads to severe mitochondrial fragmentation and cristae disorganization [7]. To assess the effects of AFG3L2 knockdown, and to compare the impact of single and combined knockdown of AFG3L2 and YME1L on mitochondrial morphology and ultrastructure, we used fluorescent mitochondrial imaging and transmission electron microscopy. MitoTracker^®^ Red CMXRos fluorescence imaging showed that, whereas mitochondria of AFG3L2 KD cells demonstrated only moderate fragmentation, mitochondria of YME1L KD, as well as of YME1L/AFG3L2 KD cells, were severely fragmented with almost a complete lack of tubular organelles and filamentous network (Figure 2A,B). The lack of severe mitochondrial fragmentation in AFG3L2 KD cells is in agreement with a recent report of AFG3L2 being required for OMA1 maturation [15]. Likewise, the partial rescue of YME1L-induced fragmentation by concurrent AFG3L2 silencing can be attributed to hampered OMA1 maturation that is likely to hinder stress-induced cleavage of L-OPA1 by matured OMA1 [15].

Transmission electron microscopy revealed prevalence of oval-shaped mitochondria with altered and attenuated cristae architecture in all three KD cell lines (Figure 2C,D). Despite moderate mitochondrial fragmentation, AFG3L2 KD cells showed the most severe cristae depletion. Indeed, the regulation of cristae morphogenesis by OPA1 was shown to be independent of its pro-fusion activity [25,26]. OPA1 is required for cristae morphogenesis, as both its disruption as well as overexpression drastically affect cristae structure [27,28]. In agreement with our previous report, YME1L KD mitochondria showed marked disorganization of relatively abundant cristae (Figure 2C,D) [7]. The marked cristae alterations found in YME1L KD cells were suggested to stem from concurrent OMA1 activation, shifting the balance to S-OPA1 [29]. Interestingly, cells that contain only L-OPA1 due to simultaneous YME1L and OMA1 inactivation show normal cristae morphology [29]. Mitochondria of our double AFG3L2/YME1L KD cells appeared to combine both ultrastructural defects, containing diminished number of disorganized cristae (Figure 2C,D).

### 2.3. Loss of AFG3L2 and/or YME1L Leads to OPA1 Upregulation, Accumulation of Short OPA1 Forms, Elevated 60 kDa Oma1, and Reduced SPG7

**Figure 3:**
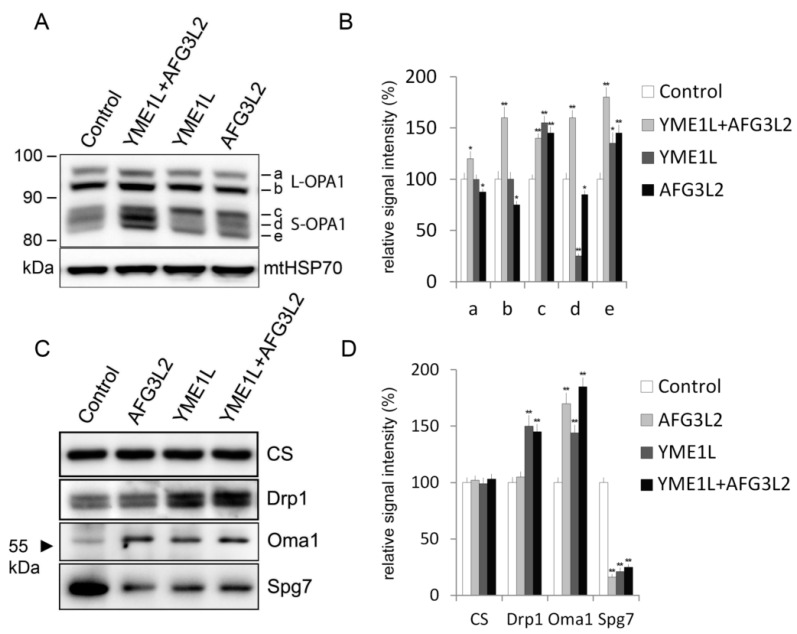
Loss of AFG3L2 and/or YME1L is associated with elevated OMA1, diminished SPG7, and a markedly altered pattern of OPA1 protein forms. Whole-cell lysates were resolved using SDS-PAGE and western blotted with antibody to OPA1 (**A**); Drp1, Oma1, and Spg7 (**C**). (**B**,**D**) The quantification of western blot signal from A and C by densitometric analysis. Western blot images representative of three independent experiments are shown. Detection of mtHSP70 and citrate synthase (CS) was used to control for equal protein loading. The values are shown as mean ± SD. * *p* < 0.05; ** *p* < 0.01.

The balance between long (L-OPA1) and short (S-OPA1) OPA1 protein forms is regulated by alternative splicing and proteolytic processing by YME1L and OMA1 zinc metallopeptidase [23]. The membrane-anchored long forms (L-OPA1) are generated upon import of the eight expressed human OPA1 isoforms by cleavage of mitochondrial targeting sequence by MPP. A fraction of L-OPA1 is then constitutively cleaved by OMA1 and YME1L proteases to produce the soluble short forms (S-OPA1) [30]. S-OPA1 band d results from cleavage of L-OPA1 by YME1L, whereas S-OPA1 bands c and e are produced by OMA1 cleavage of L-OPA1 [23,31]. We analyzed the levels of OPA1 protein forms as well as of OMA1 metallopeptidase at steady-state in whole-cell lysates of our KD cells. The OPA1 alterations found in single KD cells were mostly limited to short OPA1 forms (S-OPA1), with increase in band c and e in AFG3L2 KD cells and increase in band c and band e accompanied by reduction in band d in YME1L KD cells (Figure 3A). Knock-out of AFG3L2 in MEFs was shown to lead to severe reduction in L-OPA1 and marked reduction in S-OPA1 bands d and e [32]. In agreement with the pro-fusion activity of L-OPA1 this was accompanied by severe mitochondrial fragmentation [32]. Our AFG3L2 KD cells showed only mild reduction in L-OPA1, which was in line with the moderate mitochondrial fragmentation seen in these cells, when compared to YME1L KD mitochondria (Figure 2A,B). The discrepancy in OPA1 L-/S- forms balance may be attributed to the residual AFG3L2 protein level found in our KD cells, compared to KO MEFs, or to a presence of additional AFG3L1 isoenzyme in rodent cells [23].

The double AFG3L2/YME1L KD cells showed marked increase in all detectable OPA1 bands (Figure 3A,B). However, L-OPA1 band b and S-OPA1 bands d and e showed the most prominent increase in double KD cells (Figure 3A). Thus, the qualitative impact of simultaneous AFG3L2 and YME1L silencing toward OPA1 protein forms appeared additive, similar to its effects towards cristae structure (Figure 2C,D and Figure 3A). The observed upregulation of OPA1 may represent a compensatory response to severe mitochondrial stress [33]. However, the mechanism responsible for S-OPA1 accumulation under conditions of YME1L deficiency and hindered OMA1 activation due to simultaneous AFG3L2 knockdown remains to be addressed.

Western blotting detection of OMA1 in mitochondrial lysates of our KD cells showed the 60 kDa form of the protein elevated in all knockdown samples (Figure 3B). OMA1 can be found in mitochondria as a pre-pro-protein of 60 kDa as well as a N-terminally trimmed pro-protein of 40 kDa that subsequently undergoes autoproteolytic cleavage to generate the active form [15]. The product of this stress-induced cleavage is directly responsible for the generation of S-OPA1 by means of L-OPA1 processing [22]. Whereas YME1L is active constitutively, activity of OMA1 markedly increases after stress insults such as after mitochondrial membrane depolarization [22,34]. The markedly elevated levels of endogenous 60 kDa OMA1 found in our AFG3L2 KD cells are in accordance with a recent report identifying AFG3L2 as a protease mediating the initial OMA1-HA cleavage [15]. The study further reported a lack of accumulation of 60 kDa OMA1 upon YME1L silencing, yet showed a marked increase in the 40 kDa form of the protein under such conditions. This is partly in contrast with our results since we found increased accumulation of 60 kDa OMA1 also in cells with isolated YME1L knockdown and did not detect the 40 kDa form of the protein in these cells (Figure 3B). This difference might stem from the distinct behavior of endogenous OMA1 and ectopically expressed OMA1-HA under such conditions [15]. Moreover, since OMA1 has been shown to be degraded in a YME1L-dependent manner, its accumulation in YME1L deficient cells could be expected [10].

Dynamin-related protein 1 (Drp1) GTPase is crucial for mitochondrial fission [35]. It was reported that loss of Drp1 inhibited mitochondrial fragmentation of YME1L knockdown mouse embryonic fibroblasts (MEFs) [8]. We found increased levels of Drp1 in the mitochondrial lysates of YME1L KD and double AFG3L2/YME1L KD cells (Figure 3B). This is consistent with reported increase of the mitochondrial fission factor (Mff), mitochondrial division factor 49 (MiD49), and key DRP1 adaptors in YME1L knockdown MEFs [8] and is likely to trigger the marked fragmentation of mitochondria in YME1L KD cells (Figure 2A,B).

Whereas the human i-AAA protease is formed by homo-oligomerization of the sole YME1L subunit, the m-AAA protease exists in human cells as two isoenzymes: a homo-oligomer of AFG3L2 subunits and a hetero-oligomer of AFG3L2 and SPG7 [5]. Surprisingly, after checking the levels of SPG7 in the mitochondrial lysates of our KD cells using western blotting we found the protein markedly diminished, not only in AFG3L2 KD cells but also in cells with isolated YME1L knockdown (Figure 3B). Indeed, it was demonstrated that in MEFs, SPG7 requires one of the two mouse m-AAA isoenzymes (AFG3L2, AFG3L1) for its post-import maturation [36]. Therefore, it is possible that the failure to undergo AFG3L2-dependent maturation leads to impaired accumulation of SPG7 in the mitochondria of our AFG3L2 KD cells. However, the question of how isolated deficiency of YME1L compromises stability or expression/import of SPG7 into mitochondria remains to be addressed.

### 2.4. AFG3L2/YME1L KD Cells Show Reduced Complex I Holoenzyme and Impaired Activity of Complexes I, III, and IV

**Figure 4:**
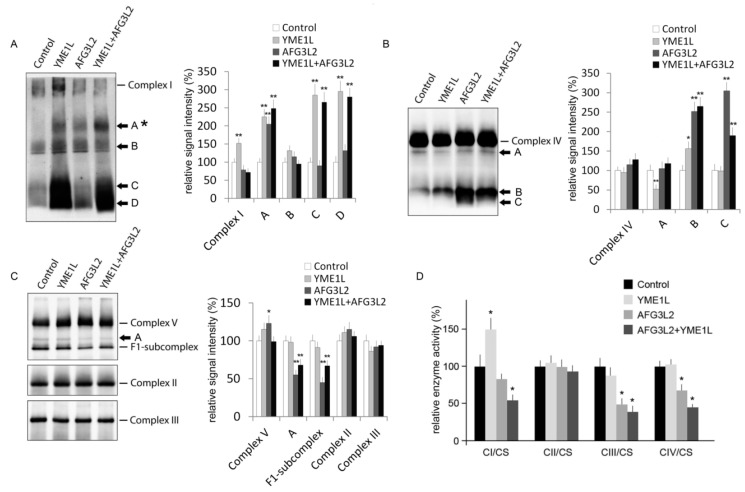
Accumulation of complex I and IV subcomplexes accompanied by impaired activity of respiratory chain complexes I, III and IV in AFG3L2, YME1L and double AFG3L2 + YME1L KD cells. Isolated mitochondrial fractions were solubilized with 1% dodecyl maltoside, and equal amounts of protein extracts were resolved using BN-PAGE and then immunoblotted with antibodies to (**A**) NDUFB6, (**B**) Cox4 and (**C**) ATPase F1-alpha, SDHA or Core 2. Western blotting signal was quantified by densitometric analysis. The values are shown as mean ± SD. * *p* < 0.05; ** *p* < 0.01. (**D**) Enzyme activities of respiratory chain complexes I–IV were measured spectrophotometrically in isolated mitochondria. The values (mean ± SD) were normalized to activity of mitochondrial enzyme citrate synthase (CS). * *p* < 0.05.

We have previously demonstrated that the i-AAA protease YME1L is involved in proteolytic control of respiratory chain biogenesis via clearance of excess or damaged NDUFB6 and Cox4 subunits [7]. Here, we used blue-native immunoblotting to analyze holoenzyme levels and assembly profiles of OXPHOS complexes in cells with loss of AFG3L2 and/or YME1L. In agreement with our previous report, loss of YME1L resulted in accumulation of NDUFB6 subunit leading to the appearance of multiple complex I subcomplexes and increased amount of complex I holoenzyme (Figure 4A) [7]. Interestingly, although the qualitative profile of Ndufb6-subcomplexes in AFG3L2/YME1L KD cells resembled that of single YME1L KD cells, complex I holoenzyme was not elevated in the double protease knockdown but significantly reduced (Figure 4A). In addition, the low molecular weight complex I subcomplexes were reduced, and the high molecular weight Ndufb6 subcomplex (marked with *) was elevated in AFG3L2/YME1L cells, when compared to the YME1L KD profile (Figure 4A). Blue-native immunoblotting of Cox4 revealed increased accumulation of Cox4-containing subcomplex(es) in AFG3L2 KD and AFG3L2/YME1L KD cells, when compared to single YME1L KD and control cells (Figure 4B). The simultaneous AFG3L2/YME1L silencing led to disappearance of the very low molecular weight Cox4 species from single AFG3L2 knockdown cells. These might thus represent Cox4 degradation products generated by YME1L in single AFG3L2 KD cells. Other alterations included moderately reduced complex I holoenzyme, as well as increased complex V holoenzyme and reduced F1-subcomplex of complex V in single AFG3L2 KD cells (Figure 4C). It was initially demonstrated that fibroblasts from patients with hereditary spastic paraplegia due to mutations in SPG7 lacking the heterooligomeric AFG3L2/SPG7 complexes have reduced complex I activity [37]. In a murine AFG3L2 knockout model, complex I and III activity defects due to diminished amount of fully assembled (holoenzyme) complexes were described [38]. In addition, knockdown of AFG3L2 by siRNA in HEK293 cells was shown to increase the amount of de novo synthesized MT-ATP6 in a metabolic labeling experiment suggesting a mechanism leading to the elevated complex V holoenzyme seen in our AFG3L2 KD cells [13].

Spectrophotometric measurements of respiratory chain (RC) enzyme activities showed markedly reduced activity of complexes I (54% of control), III (36% of control) and IV (42% of control) in the double AFG3L2/YME1L knockdown cells (Figure 4D). The RC enzyme activities were largely unaffected in YME1L KD cells (except for markedly elevated complex I activity). AFG3L2 KD cells had significantly reduced activity of complexes III and IV and moderate reduction in activity of complex I (Figure 4D). Both the elevation of complex I holoenzyme in YME1L KD cells as well as its marked reduction in double AFG3L2/YME1L cells corresponds with the elevated and markedly diminished enzyme activity of NADH:ubiquinone oxidoreductase in these cells (Figure 4D). The activity of mitochondrial marker enzyme citrate synthase (CS) did not significantly differ among the studied cell lines, which corresponds with unaltered steady-state levels of CS in these cells (Figure 3B).

Here, we have addressed the impact of isolated and simultaneous knockdown of mitochondrial m-AAA and i-AAA protease subunits towards mitochondrial structure and respiratory chain integrity. The most important findings of this work include the marked upregulation of OPA1 upon simultaneous knockdown of both AAA proteases, increased OMA1 (60 kDa) and markedly reduced Spg7 upon i-AAA protease silencing, and partial rescue of lamellar cristae morphology of AFG3L2 deficient mitochondria by concurrent YME1L silencing. Our results may help in the understanding of mechanisms of severe human neurodegenerative pathologies associated with mutations in genes encoding mitochondrial AAA protease subunits.

## 3. Materials and Methods

### 3.1. Cell Culture and Transfection

Human embryonic kidney cells (HEK293, CRL-1573) were purchased from the American Type Culture Collection (Rockville, MD, USA) and cultivated in high-glucose DMEM (Dulbecco’s Modified Eagle Medium) (PAA Laboratories, Pasching, Austria) supplemented with 10% (*v*/*v*) fetal bovine serum Gold (PAA Laboratories) at 37 °C in a 5% (*v*/*v*) CO_2_ atmosphere.

### 3.2. shRNA, ORF Constructs, and Mutagenesis

A negative control pGIPZ shRNAmir construct and pGIPZ shRNAmir constructs (V2LHS 259777, V2LHS_211676) targeting the human AFG3L2 and YME1L transcripts were purchased from Open Biosystems (GE Dharmacon, Lafayette, CO, USA). To construct stable KD cells, subconfluent HEK293 cells (10^7^) were electroporated using Nucleofector™ (Lonza, Walkersville, MD, USA) with cell specific kit according to the manufacturer’s instructions. Stable transfectants were selected with puromycin (1.5 μg/mL) over a period of 3 wk. Immunoblot analysis was employed to check the knockdown efficiency at the protein level in stable cell lines.

### 3.3. Epifluorescence and Electron Microscopy

For epifluorescence microscopy, intact HEK293 cells were stained with 10 nM MitoTracker Red CMX Ros (Molecular Probes, Eugene, OR, USA) for 15 min in phosphate-buffered saline (PBS) and viewed at 24 °C with a Nikon Diaphot 200 inverted microscope (Nikon, Tokyo, Japan) equipped with a Plan-Apochromat 60×, numerical aperture 0.95, oil objective (Carl Zeiss, Wetzlar, Germany). The images were taken with an Olympus DP50 CCD camera (Olympus, Milan, Italy) and Viewfinder Lite 1.0 software (Pixera, Santa Clara, CA, USA). For electron microscopy analysis, the cells were fixed using a modification of Luft’s method [39]. Briefly, the cells were incubated in PBS containing 2% potassium permanganate for 15 min, washed with PBS, and dehydrated with an ethanol series. The cells were subsequently embedded in Durcupan Epon (Electron Microscopy Sciences, Hatfield, PA, USA), sectioned by Ultracut microtome (Reichert, Depew, NY, USA) to thicknesses ranging from 600 to 900 Å, and finally stained with lead citrate and uranyl acetate. A JEOL JEM-1200 EX transmission electron microscope (JEOL, Tokyo, Japan) was used for image acquisition.

### 3.4. Electrophoresis and Western Blotting

Electrophoresis and immunoblotting were performed essentially as described previously [7,40], except that a semi-dry blotter FastBlot B64 (Biometra, Germany) was used for electrotransfer. BN-PAGE (Blue Native PAGE) was used with polyacrylamide 8–15 and 8–16% (*w*/*v*) gradient gels using a Mini Protean^®^ 3 System (Bio-Rad Laboratories, Hercules, CA, USA. Mitochondrial fractions were solubilized with DDM (n-dodecyl β-d-maltoside; Sigma–Aldrich, St. Louis, MO, USA) at a final DDM/protein ratio of 1.0 mg/mg in a buffer containing 1.5 M aminocaproic acid, 2 mM EDTA and 50 mM Bis-Tris (pH 7.0) at 4 °C. Serva Blue G (Serva, Heidelberg, Germany) was added to protein samples to a final concentration of 0.1 mg/mg of detergent, and 5–50 μg of total protein was loaded per lane. The electrophoretic run was carried at 40 V, 4 °C for 1 h and then at 100 V, 4 °C. Tricine SDS/PAGE was performed with 10% polyacrylamide gels, and approx. 10 μg of protein was loaded per lane. I-block solution (Applied Biosystems, Foster City, CA, USA) (0.2% *w*/*v*) was used for membrane blocking to prevent nonspecific interactions. Immunoblots were subsequently incubated with either West Femto or West Pico chemiluminescent substrates (Pierce, Rockford, IL, USA). Acquisition of the immunoblot signal was performed with the G:BOX imaging system (Syngene, Cambridge, UK). Quantification of the resulting digital images was done using the Quantity One application (Bio-Rad).

### 3.5. Antibodies

The monoclonal antibody to human AFG3L2 (Ab68023) (1:500) was obtained from Abcam, UK. The antibody to mtHSP70 (1:1000) was from Lonza, Switzerland. Antibodies to ATPase F1-α (1:1000), SDHA (1:1000), Core 2 (1:1000), NDUFB6 (1:1000), COX1 (1:1000), COX2 (1:1000), COX4 (1:1000), COX5A (1:1000), COX6A (1:500), citrate synthase (1:2000), Drp1 (ab56788) (1:500), and OMA1 (ab104316) (1:500) were obtained from Abcam, UK. The antibody to OPA1 (612607) (1:750) was purchased from BD Biosciences (Oxford, UK), and the antibody to Spg7 (NBP2-01860) (1:500) was from NOVUS Biologicals. The antibody to YME1L (1:500) was from a previous study [7,40].

### 3.6. Mitochondrial Isolation and Subfractionation

Cell disruption by hypo-osmotic swelling coupled to Dounce homogenization was used for isolation of mitochondria-enriched fractions. Removal of nuclear contamination using low-speed centrifugation (2000× *g*) and fractionation in discontinuous sucrose density gradient (1–1.5 M) using ultracentrifugation (85,000× *g*) was carried out essentially as described herein [41]. Optimal cell disruption was assessed microscopically and nuclear contamination was controlled using histone immunoblotting.

### 3.7. Assessment of Cell Proliferation

HEK293 cells were seeded in six-well plates at 5 × 10^4^ cells per well and cultured in DMEM containing 1 μg/mL puromycin. The culture medium was replaced on the second, fourth, and sixth days. Viable cells were counted every 24 h for a total of 7 d using a Scepter Handheld Automated Cell Counter (Millipore, Billerica, MA, USA). Cell viability was assessed using the trypan blue exclusion assay.

### 3.8. Assesment of Mitochondrial Morphology and Ultrastructure

Cells containing tubular or fragmented mitochondria were counted in a double-blind manner. More than 100 cells were scored per experiment. Mitochondrial structures >5 µm in length/diameter were considered tubular whereas mitochondrial structures <5 µm in length/diameter were considered fragmented. Approximately 50 EM sections of individual cells were scored in a double-blind manner. Parallel running cristae were considered lamellar, whereas extensively branched cristae and those arranged in non-parallel manner were scored as disorganized.

### 3.9. Enzyme Activity Assays

The enzyme activities of respiratory chain complexes I–IV were measured spectrophotometrically in isolated mitochondria essentially as previously described [42].

### 3.10. Statistical Analysis

All experiments were performed in at least triplicate. The Western blots shown are representative of at least three independent experiments. For analysis of significance a non-parametric Cox’s F-test suitable for lower number of cases with non-normal data distribution (either exponential or Weilbull) was employed; *p* < 0.05 was considered as statistically significant, null hypothesis was rejected and significance is denoted as follows: * *p* < 0.05; ** *p* < 0.01.
